# Standardized Implementation of Evidence-based Guidelines to Decrease Blood Transfusions in Pediatric Intensive Care Units

**DOI:** 10.1097/pq9.0000000000000165

**Published:** 2019-04-09

**Authors:** Sheila J. Hanson, Erin B. Owen, Mark J. McDonald, Katherine J. Woods, Vicki L. Montgomery

**Affiliations:** From the * Department of Pediatric Critical Care, Children's Hospital of Wisconsin/Medical College of Wisconsin, Milwaukee, WI; † Department of Pediatrics, Medical College of Wisconsin; ‡ Division of Pediatric Critical Care, Norton Children's Hospital /University of Louisville School of Medicine Louisville, KY.

## Abstract

**Introduction:**

Despite evidence that red blood cell (RBC) transfusions may be associated with more harm than benefit, current transfusion practices vary significantly. This multicenter, quality improvement study aimed to sustainably decrease the rate of RBC transfusions in pediatric intensive care units (PICUs).

**Methods:**

This 16-month prospective study included 5 PICUs. We implemented a standardized project plan including education, bedside tools, real-time reminders, and email feedback. We collected data from consecutive transfusions during pre-implementation (Phase I), postimplementation (Phase II), and post-stabilization phases (Phase III).

**Results:**

Of the 2,064 RBC transfusions, we excluded 35% (N = 729) from analysis in patients undergoing extracorporeal membrane oxygenation. Transfusion/1,000 admissions improved throughout the study periods from a baseline 209.6 -199.8 in Phase II and 195.8 in Phase III (*P* value < 0.05). There were fewer transfusions outside of the hemoglobin threshold guideline, decreasing from 81% of transfusions outside of guidelines in Phase I to 74% in Phases II and III, *P* < 0.05. Study phase, site, co-management status, service of requesting provider, admit reason, previous transfusion status, and age were associated with transfusion above guideline threshold.

**Conclusions:**

Multicenter collaboration can successfully deploy a standardized plan that adheres to implementation science principles to sustainably decrease the rate of RBC transfusion outside of guideline thresholds. However, we did not decrease the total number of transfusions in our study. The complexity of multiple specialties co-managing patients is common in the contemporary PICU. Educational initiatives aimed at one specialty may have limited effectiveness in a multifaceted system of care.

## Abstract 2

Supplemental Digital Content is available in the text.

## INTRODUCTION

Anemia and subsequent transfusion with red blood cells (RBCs) are common occurrences in critically ill children.^1,2^ Traditional teaching states that transfusing critically ill patients will augment oxygen carrying capacity and oxygen delivery, resulting in a benefit to the patient,^3^ and when untreated, severe anemia, frequently defined as hemoglobin (Hb) ≤ 5 g/dL, is associated with morbidity and mortality.^1^ Minimal data exist to show any benefit to transfusion in patients with Hb > 7 g/dL, whereas risks associated with transfusion, such as hospital-associated infection, gut ischemia, transfusion reactions, stimulation of the inflammatory response, and other events are well documented.^4–14^

Despite mounting evidence that RBC transfusions may be associated with more harm than benefit, current transfusion practices vary both within and between Pediatric Intensive Care Units (PICUs).^15,16^ Most transfusions occur at Hb values that are higher than recommended guidelines.^15^

Physicians and other healthcare clinicians are restrained in implementing evidence-based practices into their patient care. As reported in the Institute of Medicine 2001 “Quality Chasm” publication, it takes, on average, 17 years for the results of a randomized controlled trial to be implemented by the general practitioner.^17^ Pediatric intensivists have been reticent to change their transfusion practices for critically ill and injured infants and children despite over 10 years of publications describing little benefit and significant risk of RBC transfusion in critically ill children and adults.^18^

An organized approach to implementation can have a positive impact on change. Baer et al,^19^ in a 2011 publication, reported that a system-wide implementation of a neonatal transfusion reduction compliance program resulted in improved compliance with transfusion guidelines from 65% to 90%. Their strategies included involvement of bedside providers in drafting and implementing the transfusion guidelines, requirement of ordering all blood products through computerized order entry, and requiring a reason for any transfusion ordered outside of the guidelines. The percentage of patients receiving a transfusion decreased from 19% to 13% and a savings of $469,238 per year occurred.

Thus, the goal of this multi-institutional, quality improvement study was to evaluate whether a standardized implementation plan, across multiple US PICUs, can lead to a sustainable decrease in the rate of RBC transfusions ordered in PICUs.

## METHODS

### Context and Setting

This multisite PICU collaborative included 5 centers from the National Association of Children’s Hospitals (now known as Children’s Hospital Association) PICU Focus Group. Each site had a pediatric intensivist, at least one PICU nurse, and the site Blood Bank Medical Director involved in the study. All sites received local Institutional Review Board approval before data collection.

### Study Periods

**Fig. 1.**
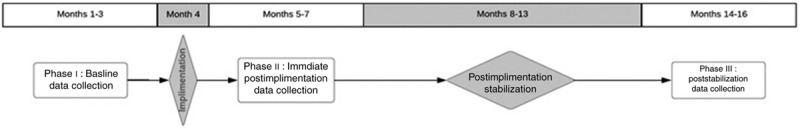
Study timeline.

This project was a prospective, quality improvement study. No order for transfusion was withheld or required because of this study. We divided this 16-month study into the following periods (Fig. 1).

1. Phase I (months 1–3): a 3-month preimplementation phase
  - Data collection: baseline transfusion practices;
  - Local buy-in and leadership initiation;
  - Planning, Educational, and Quality Management strategies as listed in the Implementation Strategies section.
2. Implementation interphase (month 4): 1 month with no data collection for deployment of the standardized implementation plan
  - Training session for site Principal Investigators (PIs) via webinar and regularly scheduled Children’s Hospital Association PICU Focus Group Meetings (Focus Group);
  - Slide presentation of transfusion evidence to each PICU section;
  - Dissemination and attestation of “Must read” articles;
  - Identification of nurse champions with training of bedside nurses in the use of bedside transfusion aid;
  - Educational strategies as listed in the Implementation Strategies section.
3. Phase II (months 5–7): a 3-month immediate postimplementation phase, to determine the immediate impact of the implementation plan on transfusion practices
  - Data collection: postimplementation transfusion practice;
  - Bedside nurse provides verbal reminders when the order is for a patient with a Hb > 7 g/dL (greater than 9.5 g/dL in children with baseline cyanosis);
  - Email to individual pediatric intensivist each time transfusion occurs when patient Hb > 7 g/dL (greater than 9.5 g/dL in children with baseline cyanosis) to serve as a reminder that if evidenced-based criteria for transfusion were not present, which the literature does not support transfusion;
  - Blinded aggregate summary of all participating PICU transfusion data provided to each site during Focus Group meetings for local dissemination;
  - Unit summary and blinded individual provider feedback by email no less frequently than every 2 weeks. The site PI knew prescriber identity at each site, but we presented data in a blinded fashion. Each prescriber knew his/her data but not the unblinded practices of the other members of the group;
  - Quality Management Strategies as listed in the Implementation Strategies section.
4. Stabilization interphase (months 8–13): 6 months with no data collection or feedback to participants.
5. Phase III (months 14–16): a final 3-month poststabilization phase to assess sustained change in transfusion practices
  - Data collection: Sustained postimplementation transfusion practice;
  - No standardized prompting or feedback during this phase.

Study initiation and data collection occurred independently at each site.

### Implementation Strategies

Mapping our implementation strategies to the compilation of strategies reported by Powell et al,^20^ for implementing clinical initiatives in healthcare, we used a combination of Planning, Education, and Quality Management Strategies. Planning strategies in the prestudy development and Phase I included: gathering information, developing a formal implementation blueprint, tailoring strategies to site-specific delivery, building buy-in with local consensus discussions, identification and preparation of champions, building a team coalition, and partnership with nursing and nursing leadership. These early phases also included Educational Strategies (developing and distributing effective educational materials, conducting educational meetings, and ongoing training of site PIs) and Quality Management Strategies (developing tools for quality monitoring and using advisory boards and workgroups). The implementation interphase included predominately educational strategies: developing educational materials tailored for a local site, conducting educational meetings at each site, and conducting ongoing training of pediatric intensivists, fellows, nurse practitioners, and bedside nurses. Phase II, immediate postimplementation phase, relied on quality management strategies: auditing and providing feedback, reminding clinicians, capturing and sharing local knowledge, and organizing clinician implementation meetings. The remaining stabilization interphase and Phase III (sustained stabilization phase) had no active implementation strategy but consisted of behind-the-scenes data collection to assess the sustained effectiveness of the prior implementation strategies.

### Standardized Implementation Plan

**Fig. 2.**
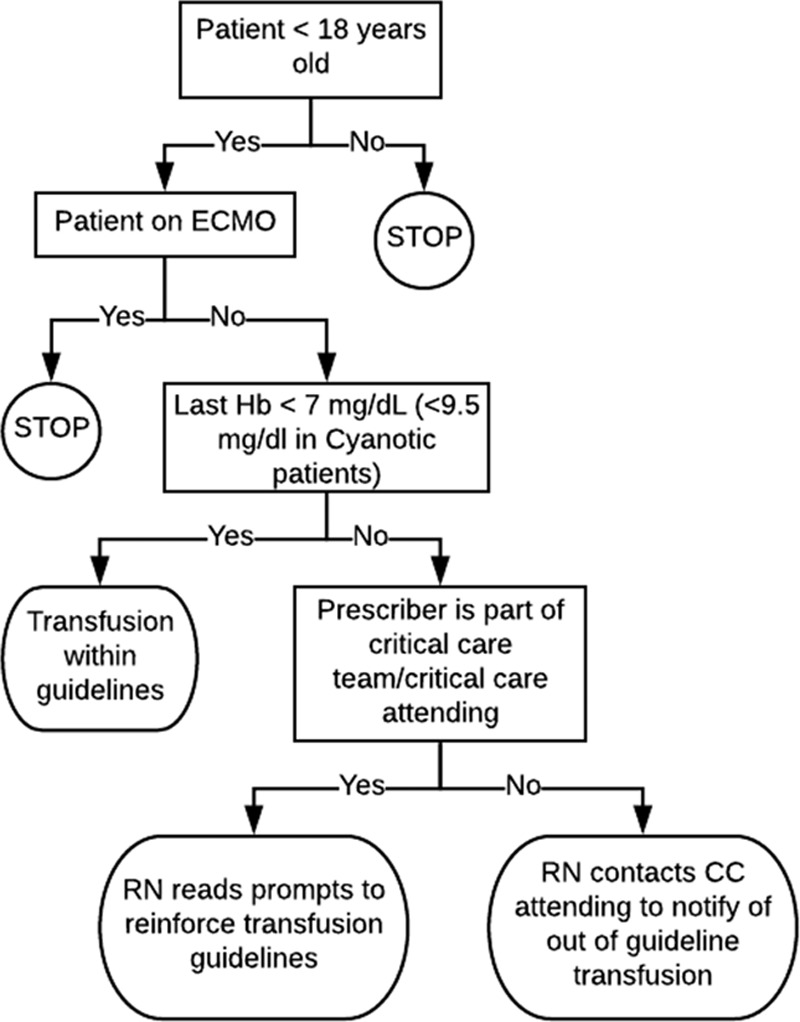
Bedside algorithm. CC, critical care; RN, registered nurse.

During the implementation interphase, study month 4, the standardized implementation plan (Table 1) was put in place by the PI at each site to reinforce evidence-based transfusion guidelines (at or below Hb of 7 g/dL or Hb of 9.5 g/dL for patients with baseline cyanosis). The plan included education, distribution of bedside tools, and collection of attestation sheets for the “must read” list (see **Appendices A**–**C, Supplemental Digital Content**, available at http://links.lww.com/PQ9/A78). The site PI provided uniform quality improvement transfusion education for the pediatric intensivists, registered nurses, advanced practice nurses, fellows, and physician assistants. Pediatric intensivists and critical care fellows were requested to read the “Must Read” articles and turn in the attestation sheet (see **Appendix A, Supplemental Digital Content**, available at http://links.lww.com/PQ9/A78). Nurses in the PICU were given a bedside algorithm (Fig. 2) to use as an aid to remind pediatric intensivists of the study when ordering blood outside of guidelines. Study investigators emailed updates of transfusions outside of guideline threshold to attend providers periodically during Phase II (see **Appendix C, Supplemental Digital Content**, available at http://links.lww.com/PQ9/A78).

**Table 1.**
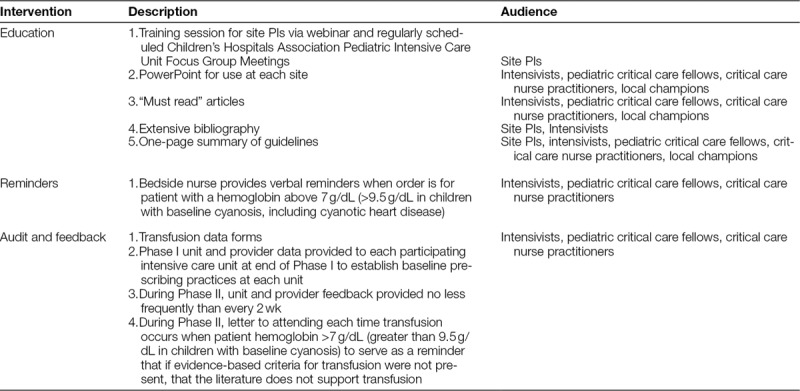
Components of the Standardized Transfusion Implementation Plan

No standardized prompting or feedback was completed during the postimplementation stabilization phase (months 8 and 13).

### Data Collection Phases

During Phase I (preimplementation), Phase II (postimplementation), and Phase III (poststabilization), we collected the number of patient admissions, patient days, and total RBC transfusions including detailed data from consecutive transfusions during each phase. No data were collected during the implementation (month 4) or postimplementation (months 8 through 13) interphases.

### Transfusion Data

We defined each RBC transfusion episode in PICU patients younger than 18 years of age as the administration of packed RBCs associated with one Blood Bank Transfusion Record. The local study team entered deidentified data from each transfusion episode into a REDCap (Research Electronic Data Capture) Database (Nashville, Tenn.) maintained at the Medical College of Wisconsin. We collected factors hypothesized to influence the decision to transfuse on all nonextracorporeal membrane oxygenation (non-ECMO) transfusions: demographics, comanagement status, service of provider ordering transfusion, the reason for admission, and previous transfusion status. Comanagement was present for any patient whose ICU care was managed by additional, noncritical care physicians at the time of ordering the transfusion. A simple consult was not considered comanagement. Comanaging services may or may not write orders on patients and are hypothesized to have varying influence on the decision to transfuse.

Prior transfusion included any transfusion received by the patient during the current PICU stay, excluding those ordered in the emergency department (ED) and/or operating room (OR). Baseline cyanosis included patients who had expected SpO_2_ < 88% when at baseline health. Most of these patients had cyanotic heart disease, but the definition did not exclude other etiologies of cyanosis. We excluded transfusions of patients on ECMO as the current guidelines do not address this population and many unique factors drive transfusions while on ECMO.

If in the 24 hours before receiving the transfusion, the subject did not have a Hb of ≤ 7 g/dL (or ≤ 9.5 g/dL for those with baseline cyanosis), the transfusion was above the guideline threshold. We collected additional data from the electronic health record of these patients to evaluate for other factors influencing the decision to transfuse. These data included autologous or directed donor status, renal replacement therapy, blood priming of dialysis circuit, presence of hypoxemic respiratory failure with FiO_2_ >60%, positive end-expiratory pressure (PEEP) > 10 mm Hg, or oscillatory ventilation at time of transfusion, baseline cyanosis with expected SpO_2_ < 88%, evidence of shock, continuous infusion of vasoactive medications, acute bleeding, or preoperative transfusion requested by anesthesia or surgical team. Circumstances that qualified as acute bleeding included: >5 mL/kg/h of blood loss over 4 hours preceding the transfusion (estimated); >10 mL/kg/h of blood loss averaged during the hour preceding the transfusion (estimated); or bleeding that is unable to be quantified (more bleeding than anticipated) with increase in heart rate and decrease in blood pressure.

### Data Analysis

Categorical variables are presented as the frequency with the corresponding percentage. Continuous variables are presented as mean (SD) or median (interquartile range) as appropriate depending on the distribution of the data. A Chi-square test or a Fisher’s exact test was used to examine the difference or relationships between categorical variables. A Mann–Whitney test was used to test the difference continuous or ordinal variables between groups.

We performed bivariate and multivariate logistic regression using the Poisson analysis on the final data to evaluate whether the standardized implementation plan is an independent factor of the transfusion event after being adjusted to other factors. We calculated the adjusted odds ratio of the event for the primary factor.

## RESULTS

There were 2,064 RBC transfusions among the 5 sites during the study period. Thirty-five percent (N = 729) of transfusions were for patients undergoing ECMO and excluded from analysis. Table 2 shows transfusion rates and patient factors. The number of transfusions did not significantly change throughout the study. However there were fewer transfusions outside of guideline Hb threshold decreasing from 81% of transfusion outside of guidelines in Phase I to 74% in Phases II and III, *P* < 0.05. Accordingly, guideline adherence increased, from 20% of transfusions being below the guideline Hb threshold in Phase 1 increasing to 26% in Phases II and III. Table 3 summarizes additional risk factors for patients transfused above the guideline threshold (Hb > 7 g/dL or Hb > 9.5 g/dL for cyanotic patients). Prevalent factors included: patients with baseline cyanosis (35%–44%), shock (29%–33%), and receipt of continuous vasoactive infusion (53%–59%). Twenty-two percent (219/1,018) of patients transfused above threshold had no identified risk factors.

**Table 2.**
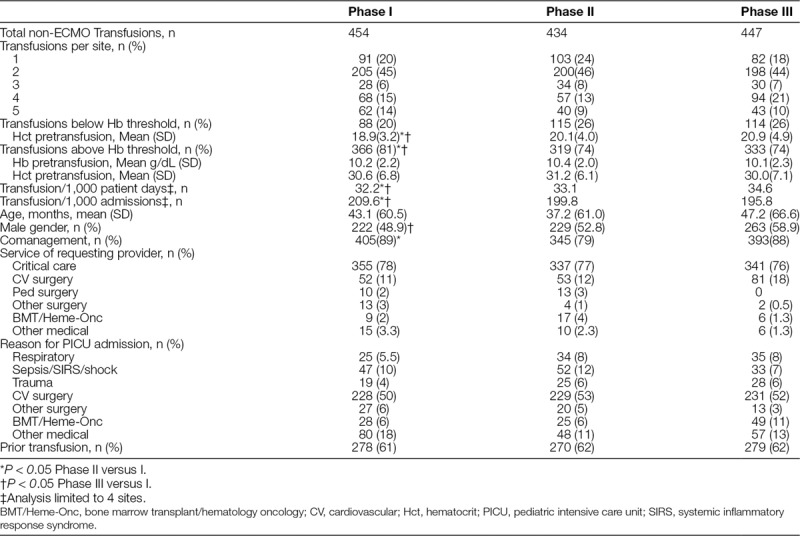
Transfusion and Patient Details by Phase

**Table 3.**
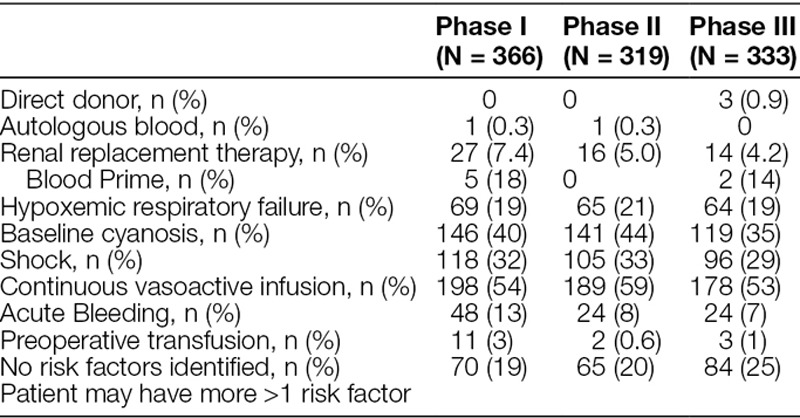
Patient Factors Associated with Transfusion above Threshold (Hb > 7 Acyanotic, Hb > 9.5 Cyanotic)

Transfusions per 1,000 patient admissions and 1,000 patient days are shown in Table 2 by the phase of the study. Analysis of these 2 rates was limited to 4 sites given missing data from one site. Transfusion decreased per 1,000 patient admissions from Phases III versus I and Phases II versus I, with no significant differences between Phases III and II. Multivariate logistic regression analysis found the phase of the study, site, service of ordering provider, and Hb (*P* < 0.05) to be significantly associated with transfusion per 1,000 patient admissions. Transfusion increased per 1,000 patient days from Phases III versus Phase I and Phases II versus I, with no significant difference between Phases III and II. Multivariate logistic regression analysis found the phase of the study, site, service of ordering provider, Hb, and gender (*P* < 0.05) to be associated with transfusion per 1,000 patient days.

When analyzed by site and phase of the study, transfusion per 1,000 patient days shows different patterns by site, with some sites obtaining an early decrease in transfusion rate, others with a delayed decrease, and others with no change or an increase in transfusion rate after implementation. Two sites showed an immediate drop in transfusion rate per 1,000 patient admissions after implementation; however, only one of these sites sustained the decreased rate. Three sites not only sustained the decrease in rate/1,000 admissions, but continued to decrease throughout the sustainability Phase III (data not shown).

The bivariate analysis determined that study phase, site, comanagement status, provider service, admit reason, previous transfusion status, and age were associated with transfusion above versus below the guideline threshold. This model found Phases II versus I and Phases III versus I to be associated with significantly increased odds of transfusion within-guideline thresholds. There was no difference between Phases III and II (Table 4). After multivariate analysis, incorporating significant predictor variables from the bivariate analysis, the study phase was no longer associated with transfusion practice.

**Table 4.**
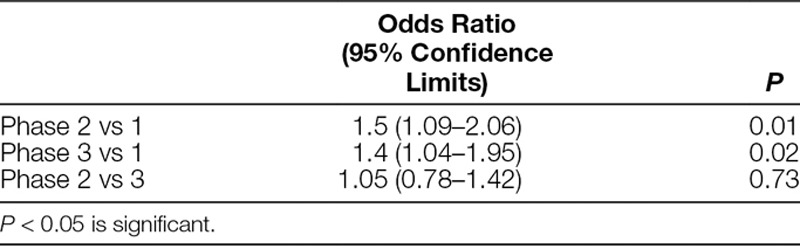
Bivariate Analysis of Transfusion within Threshold (Hb < 7 g/dL for Acyanotic Patients, Hb < 9.5 g/dL for Cyanotic Patients)

## DISCUSSION

This multicenter quality improvement study implemented a multipronged standardized education plan, including bedside, prompts, and real-time email feedback, to sustainably reduce the rate of RBC transfusions outside of evidence-based thresholds. Although there was no significant difference in a total number of transfusions before and after implementation, there was a 7% decrease in the percent of transfusion outside of the Hb threshold (above 7g/dL for acyanotic children and 9.5 g/dL for those with baseline cyanosis), after implementation.

Previous studies, including the landmark TRIPICU study,^5^ excluded patients with hemodynamic instability (hypotension or recent change in vasopressor support) or active bleeding. To capture the complex patient population and decision-making surrounding transfusion in children in the ICU, additional factors indicative of hemodynamic instability, including active bleeding, hypoxic respiratory failure, and need for vasoactive infusions, were collected for those patients transfused above the guideline thresholds. As the existing evidence is based upon patients with relatively stable hemodynamics (vasopressor support without escalation, for example), generalization to actively decompensating or unstable patients may not be appropriate and was not the focus of this study. We collected the additional factors to characterize better the type of patients receiving RBC transfusion in ICUs. Consistent with a critically ill population, 75%–81% of the transfusions were in patients with one or more of these additional factors (Table 3), most commonly the use of vasoactive infusions. There were no additional factors identified in almost 20% of the transfusions outside of the guidelines. During the study design, the authors speculated that this group with no additional factors identified would be the most likely to show improved compliance after study implementation. However, the percentage of transfusions outside of the guidelines attributed to this “low risk” did not change throughout the study.

In our study, the rate of transfusions per 1,000 patient admissions showed improvement between Phase 1 and Phases 2 and 3, but the opposite was true when we considered the rate of transfusion per patient days. The total patient admissions increased from Phases I, II, and III, whereas the total patient days decreased throughout the study. The different denominators used to calculate the transfusion rates results in the varying direction of change of transfusion rates.

The decision to transfuse is a complicated process, and as found in our study, incorporates multiple medical and surgical services as well as numerous patient factors. Over 80% of children receiving RBC transfusion in our study were comanaged with another service. Education initiatives aimed at one specialty, in this case, pediatric critical care, may have limited effectiveness in such a multifaceted system of care.

Despite guidelines for transfusion administration and supporting evidence, the study results suggest intensivists believe critically ill patients benefit from higher hemoglobin concentration, especially when their course is complicated with cyanosis or the need for vasoactive support. After excluding 35% of transfusions for patients on ECMO, patients with an admission reason of cardiovascular surgery account for 50% of the transfusions throughout all phases of the study. Also, 35%–45% of the patients receiving transfusion above the guideline threshold (Hg > 9.5g/dL) had baseline cyanosis, most commonly associated with congenital heart defects. This pediatric cardiac population is at high risk for blood transfusion and would benefit from a separate study.

The 5 sites included in this study varied in size and geographical region of the country, with one site contributing almost half of the transfusions. The qualitative pattern of change in transfusion practice throughout the study varied by the individual site (data not shown). It is unclear what factors contributed to the differences in successful implementation of transfusion guidelines but may include study team composition, hospital culture, and patient or provider-specific factors.

Implementation strategies are essential to disseminate and incorporate evidence-based treatments into healthcare. Clear description and reporting of strategies allow measurement of effectiveness and reproducibility,^21^ and so we have attempted to provide the reader with a rather detailed report of specific implementation strategies (**Methods and Appendices A**–**C**, **Supplemental Digital Content**, available at http://links.lww.com/PQ9/A78). We were not able to assess the effectiveness of the individual components of our multifaceted implementation strategy study design. For example, we did not formally measure the reception of provider feedback during the study, nor the change in practice after feedback. Anecdotally per site PIs, the feedback was received neutrally to positively, varying by individual and site. Also, we did not collect the specific details of each implementation strategy with this study. For example, although each site consistently used the Planning Implementation strategy of identification and preparation of nurse champions, the number of people recruited and the duration, frequency, and details of preparation were left to the discretion of the local site investigator. These differences in implementation may have contributed to site variation in transfusion practice.

Further limitations of this study include those of any implementation project; multiple changes may occur during the 16-month study time frame that could confound the results including a change in providers, patient census and acuity and competing hospital mandates. Additionally, the larger volume of transfusion data contributed by one site may have skewed the results disproportionately.

Strengths of this quality improvement initiative include collaboration between multiple children’s hospitals; prospective, robust data collection of important clinical variables, and a study phase focused on the sustainability of the initial results of implementation.

## CONCLUSIONS

Multicenter collaboration can successfully implement standardized education to decrease and sustain the rate of RBC transfusion outside of guideline thresholds. However, we did not decrease the total number of transfusions in our study. The complexity of multiple specialties comanaging patients is common in the contemporary PICU. Educational initiatives aimed at one specialty, in this case, pediatric critical care, may have limited effectiveness in such a multifaceted system of care.

## ACKNOWLEDGMENTS

We thank Gloria Larson, National Association of Children’s Hospitals (now known as Children’s Hospital Association) PICU Focus Group for her ongoing collaboration and support, and Chunhong Bai of Children’s Hospital Association for statistical support. We thank all the investigators and research coordinators for their critical contributions to this study: Akron Children’s Hospital, Akron, OH: Jean Christopher, RN, MSN; Ann Marie Brown, RN, PhD; Children’s Hospital Los Angeles, Los Angeles, CA: Sherry Cauley, RN, BSN; David Schmidt, RN, MSN; Children’s Hospital and Medical Center, Omaha, Nebraska: Luke Noronha, MD; Children’s Hospital of Wisconsin, Milwaukee, WI: Mary Kasch; INOVA Children’s Hospital, Fairfax, VA: William Stotz, MD; Gail Green.

## DISCLOSURE

The authors have no financial interest to declare in relation to the content of this article.

## Supplementary Material
