# Supplementary material for: Standardized Implementation of Evidence-based Guidelines to Decrease Blood Transfusions in Pediatric Intensive Care Units
Source: Pediatr Qual Saf. 2019 Apr 9;4(3):e165. doi: 10.1097/pq9.0000000000000165 (PMC6594784; doi:10.1097/pq9.0000000000000165)
Supplement: Supplementary file 1 [file pqs-4-e165-s001.docx]

Supplementary Digital Content

Appendix A: Must Read Articles and Attestation

| **"Must Read Articles" Attestation** | | | |
| --- | --- | --- | --- |
| Attending Intensivist Name |  | | |
| Author | Article | | |
| Bateman | Anemia, Blood Loss, and Blood Transfusions in North American Children in the Intensive Care Unit | I have read this article. | ☐ Yes ☐ No |
| Cholette | Children with single-ventricle physiology do not benefit from higher hemoglobin levels post cavopulmonary connection: Results of a prospective, randomized, controlled trial of a restrictive versus liberal red-cell transfusion strategy | I have read this article. | ☐ Yes ☐ No |
| Armano | Determinants of red blood transfusions in a pediatric critical care unit: A prospective, descriptive epidemiological study | I have read this article. | ☐ Yes ☐ No |
| Gauvin | Acute tranfusion reactions in the pediatric intensive care unit | I have read this article. | ☐ Yes ☐ No |
| Istaphanous | Red blood cell transfusion in critically ill children: A narrative review | I have read this article. | ☐ Yes ☐ No |
| Lacroix | Transfusion Strategies for Patients in Pediatric Intensive Care Units | I have read this article. | ☐ Yes ☐ No |
| Shander | Activity based costs of blood transfusion in surgical patients at four hospitals | I have read this article. | ☐ Yes ☐ No |
| Hebert | A multicenter, randomized, controlled clinical trial of transfusion requirements in critical care | I have read this article. | ☐ Yes ☐ No |
| Goodman | Pediatric red blood cell transfusions increase resource use | I have read this article. | ☐ Yes ☐ No |
| Willems | Comparison of red blood cell transfusion strategies after pediatric cardiac surgery: a subgroup analysis | I have read this article. | ☐ Yes ☐ No |
|  |  |  |  |
