# Supplementary material for: Standardized Implementation of Evidence-based Guidelines to Decrease Blood Transfusions in Pediatric Intensive Care Units
Source: Pediatr Qual Saf. 2019 Apr 9;4(3):e165. doi: 10.1097/pq9.0000000000000165 (PMC6594784; doi:10.1097/pq9.0000000000000165)
Supplement: Supplementary file 2 [file pqs-4-e165-s002.docx]

Supplementary Digital Content Appendix Table B

Bedside data collection tool for transfusion prescriber. To be completed at time of transfusion.

| **Bedside Data Collection Tool** | | |
| --- | --- | --- |
| PICU Attending: |  | |
| Patient co-managed by another service | - Yes   Other Service(s) | - No |
| In shock at time transfusion ordered? | - Yes | - No |
| Did another service request the transfusion? | - Yes   Which Service(s) | No |
| Primary reason for PICU admission? (Respiratory, Sepsis/SIRS/Shock,  Trauma, CV Surgery, Other Surgery, BMT/Heme-Onc, Other Medical |  | |

*PICU- pediatric intensive care unit, SIRS-systemic inflammatory response syndrome, CV-cardiovascular, BMT/Heme-Onc- Bone Marrow Transplant/Hematology-Oncology*
