# Supplementary material for: Standardized Implementation of Evidence-based Guidelines to Decrease Blood Transfusions in Pediatric Intensive Care Units
Source: Pediatr Qual Saf. 2019 Apr 9;4(3):e165. doi: 10.1097/pq9.0000000000000165 (PMC6594784; doi:10.1097/pq9.0000000000000165)
Supplement: Supplementary file 3 [file pqs-4-e165-s003.docx]

Supplemental Digital Content Appendix C: Letter to Attending to provide reminder of study and transfusion thresholds. Sent during Phase II when order for blood is outside recommended evidence.

Dear Dr. ______________________

During the last 2 weeks, _______ (number of transfusions outside EBM recommendations) of the _____ total number of transfusions that were ordered by you were outside the guidelines.

Based on current data, most patients in the PICU with a hemoglobin greater than 7 g/dL (greater than 9.5 g/dL in children with cyanotic heart disease) will not benefit from transfusion yet will incur the risks associated with transfusion. Although we know there are times when transfusion, even though the hemoglobin is higher, is indicated, I just wanted to provide a reminder regarding the current literature.

If you have any questions or concerns, please feel free to contact me at ___________.

Sincerely,

Site PI with electronic signatures from Project and other site PIs.
